# On the Locus of the Practice Effect in Sustained Attention Tests

**DOI:** 10.3390/jintelligence7020012

**Published:** 2019-06-04

**Authors:** Iris Blotenberg, Lothar Schmidt-Atzert

**Affiliations:** Department of Psychology, Philipps-University of Marburg, Gutenbergstr. 18, 35032 Marburg, Germany; schmidt-atzert@uni-marburg.de

**Keywords:** sustained attention, concentration, practice effects, process model, experimental test validation

## Abstract

The present study set out to explore the locus of the poorly understood but frequently reported and comparatively large practice effect in sustained attention tests. Drawing on a recently proposed process model of sustained attention tests, several cognitive tasks were administered twice in order to examine which specific component of test performance benefitted from practice and to which extent. It was shown that the tasks representing the three sub-components of sustained attention tests, namely the perception of an item, the simple mental operation to solve an item, and the motor reaction to indicate a response to an item, benefitted from practice. Importantly, the largest practice gain was observed for the task that required item-solving processes in addition to perceptual and motor processes. Two additional postulated mechanisms in sustained attention tests—the deliberate shifting between items and the preprocessing of upcoming items—did not become more efficient through practice. Altogether, the present study shows that the practice effect in sustained attention tests seems to be primarily due to faster item-solving processes and, to a limited extent, due to a faster perception of the item, as well as a faster motor response. Moreover, besides the sub-components, it is likely that also the coordination of perceptual, item-solving, and motor processes benefitted from practice. Altogether, the present paper may have taken a first step towards a better understanding of the specific processes that cause the large practice gains in sustained attention tests.

## 1. Introduction

The ability to sustain mental focus over extended periods of time, namely sustained attention (or concentration), is crucial for various everyday tasks. Accordingly, the assessment of sustained attention is relevant in many different psychological domains like neuro-, clinical or traffic psychology, as well as in personnel selection [1]. Sustained attention tests’ excellent psychometric properties have been demonstrated in multiple studies [2,3,4,5]. However, there are, like in other cognitive ability tests, substantial practice effects in this group of tests, for which the causes are largely unknown [6,7]. The aim of the present study was to explore the locus of the practice effect in these tests by drawing on a recently proposed process model of sustained attention tests.

### 1.1. Practice Effects in Sustained Attention Tests

Practice or retest effects—that is a test score gain after prior exposure to the same or an alternate form of a test under comparable conditions [8]—is a well-documented phenomenon for cognitive ability tests [9,10,11]. A recent meta-analysis [10] revealed practice effects of a third standard deviation for cognitive ability tests in general and a slightly higher practice effect of .37 standard deviations for tests of processing speed, which are conceptually similar and empirically indistinguishable from sustained attention tests [5,12]. Other studies not considered in the meta-analysis reported even larger practice effects between half and one standard deviation from the first to the second test administration [7,13]. For the German sustained attention test d2, which was applied in the present study, practice effects ranged from two-thirds standard deviation for the paper–pencil version to four-fifths standard deviation for the latest electronic version [7,14,15]. Moreover, several studies showed increasing practice effects over several test administrations. For example, after eleven test administrations, Westhoff and Dewald [13] revealed large practice effects of nearly three standard deviations for a figural and around two and a half standard deviations for a numerical sustained attention test. Even after these many repetitions, though test scores increased more slowly, they had not reached a plateau yet (see also [16]). Additionally, while retest effects have been shown to decline with longer retest intervals, they have also been reported to decline rather slowly, so much so that it takes five years for them to vanish [10]. Thus, practice effects in cognitive ability tests in general and in sustained attention tests in particular deserve consideration. Importantly, retesting is quite common in many contexts like personnel selection or the educational sector as well as in neuropsychology [10] and it impacts the validity of the task [8,10,17].

Though various factors are being discussed [8,9,18], the causes and locus (that is, the specific processes that become more efficient through practice) of the practice effect in sustained attention tests are not comprehended in detail [6,16]. In fact, there is a paucity of studies that investigate how retesting affects the specific processes and mechanisms involved in these tests. In the present study, we address this knowledge gap by focusing on the components that have been shown to drive performance in sustained attention tests. That is, using an approach of experimental test validation, we examine which of these components benefit from practice and to which extent. In the following, the characteristics of sustained attention tests and a recently proposed process model are introduced.

### 1.2. A Process Model of Sustained Attention Tests

For more than a hundred years now, sustained attention has been measured using simple, homogenous stimuli and comparatively easy tasks, like letter cancellation, simple mental arithmetic, or sorting according to categories [1,19]. Importantly, even more critical than the task itself is the typical presentation mode of these tests—that is, many stimuli are presented at the same time, and the participants are required to continuously work and respond to them until the test is over [1,20,21]. As a main indicator of performance, the number of marked items (speed) or the number of correctly marked items (error-corrected speed) is assessed [5]. 

Blotenberg and Schmidt-Atzert [22] recently proposed a process model of sustained attention tests. They argued that the operations required in these tests on the item-level are comparatively straightforward. Independent of whether the task requires the cancellation of targets, mental arithmetic or the sorting according to categories, they all require a fast *perception* of the item, which is followed by a *simple mental operation* to solve the item and a *motor response*. Additionally, according to Blotenberg and Schmidt-Atzert [23], the model is to be extended when considering the characteristic presentation mode of sustained attention tests, one that demands the deliberate, self-paced shifting between many simultaneously presented items [1,20,21]. This characteristic presentation mode should require the test-taker to shift from one item to another as quickly as possible (*item shifting* [1,20,21,24,25]). Moreover, it should also demand the test-taker to *focus* on the currently relevant item while ignoring the surrounding items (see also [26,27]). Finally, the simultaneous presentation of many items might also provide the opportunity to *preprocess* upcoming items in order to prepare for upcoming actions (see also [28,29]).

In two studies that were partly based on the same data as the current study, it was demonstrated that *perceptual, mental operation* and *motor speed* already explained a large amount of variance in conventional sustained attention tests, namely 55–74% [22]. Importantly, *perceptual* and *mental operation speed* were the strongest predictors of test performance, while there was a consistent trend towards a minor influence of *motor speed*. Moreover, while there were considerable *item shifting costs* for the deliberate, self-paced shifting between items compared to a presentation mode which involved short intervals between successive items (also called the force-paced mode), these were not related to performance in sustained attention tests. Additionally, there was no effect of *focusing*; that is, performance did not significantly differ between conditions which should have induced higher versus lower focusing demands [23]. Thus, it seems that the manipulation of an increased focusing demand in the modified d2 was not successful and therefore, it will not be further examined in the current study. Finally, the authors found a large preview benefit; that is, performance was considerably facilitated when the test-takers received the opportunity to preprocess upcoming stimuli. This *preprocessing* component proved to be substantially correlated with performance in sustained attention tests [23]. 

### 1.3. The Present Study

Drawing on these findings, the aim of the present study was to more closely investigate the practice effect in sustained attention tests and more specifically, to examine which sub-components benefit from the repeated test administration. To assess practice effects of *perceptual, mental operation* and *motor speed*, several cognitive tasks were selected so that they would successively demand additional sub-components: The inspection time task was administered as a prototypical measure of *perceptual* speed [30,31,32]. The simple reaction time task, which is considered to impose mainly motor [33,34,35] but also basic perceptual demands [36] was applied to assess *motor speed*. Additionally, a simple version of the modified d2, in which only single stimuli were presented one after another including short breaks, was applied to measure *mental operation speed* beyond perceptual and motor processes. All of these tasks were applied twice and we expected substantial practice gains for at least one of the three tasks and the respective sub-components. 

Furthermore, we explored how retesting affected the *item shifting* and *preprocessing* mechanisms. In other words: Is the large practice effect in sustained attention tests (partly) due to a more efficient *shifting between items* or *preprocessing of upcoming items* in the repeated test administration? To address this research question, further modified versions of the d2 were created and administered twice. It was examined how practice affected performance in the different versions with varying pace (self-paced versus force-paced) and stimulus arrangements (single, blocks versus rows of stimuli). For an effect of practice on *item shifting,* we should find an interaction of practice and pace so that the difference between the self-paced and the force-paced conditions would shrink through practice. Such an interaction would suggest that practice reinforced the participants’ capability to handle the intentional *item shifting* required when they work through the test in a self-paced manner. Additionally, if practice reinforced *preprocessing*, we should observe a significantly larger practice effect for the conditions with rows of stimuli (which allowed preprocessing) compared to the conditions with single or blocks of stimuli (which did not allow preprocessing). 

## 2. Method

### 2.1. Participants

One hundred undergraduates (72 female, 42 studied psychology) with a mean age of 22.9 years (*SD* = 4.6, *range* = 18–40), participated in the present study and received partial course credit in exchange. They gave informed consent prior to participation in accordance with the Declaration of Helsinki. 

### 2.2. Measures

#### 2.2.1. Perceptual Speed—The Inspection Time Task

In the inspection time task, a Pi-shaped figure with two legs of markedly different lengths is presented, and the participant is required to perceive and indicate which of the two lines is longer [37]. Based on the response accuracy, the figure exposure time is adjusted adaptively in order to assess the shortest time necessary to correctly perceive it. The inspection time task is considered a primarily perceptual task [30,31,32], and performance in this task was utilized as a measure of the sub-component of perceptual speed. The present version of the task was programmed in E-Prime 2.0 [38]. Each trial started with a white fixation cross against a black background (1000 ms). It was followed by a Pi-shaped figure with one shorter (3.5 cm) and one longer leg (4.5 cm). Afterwards, the legs were covered by a backward mask to prevent processing from stored traces (300 ms). The subject’s task was to indicate which leg was longer by pressing “c” for the left leg (with the left index finger) and “m” for the right leg (with the right index finger) on a German QWERTZ computer keyboard. The exposure time of the Pi-shaped figure was varied using a staircase procedure, i.e., four correct responses led to a shortened exposure time and an incorrect response led to an increase in exposure time. After three practice trials, the staircase procedure started with an exposure time of 157 ms and decreased or increased in steps of 66 ms (at the beginning) to 16.5 ms (in the course of the experiment). The experiment ended after 15 reversals (i.e., when the exposure time suddenly increased after it had been decreasing before or vice versa) or a maximum of 96 trials. The individual inspection time was the shortest exposure time to which the participant answered correctly for four times in a row. 

#### 2.2.2. Motor Speed—The Simple Reaction Time Task

As a prototypical measure of motor speed, a simple reaction time task was applied [33,34,35]. Participants were instructed to press the button “c” (20 trials, left index finger) and, afterwards, “m” (20 trials, right index finger) as fast as possible as soon as a black dot (2 × 2 cm) appeared on screen. The dot was presented until a response was made, and the next dot appeared after 1000 ms (+/- 100 ms jitter). The simple reaction time task consisted of 40 experimental plus 10 practice trials. The dependent variable was the mean reaction time. 

#### 2.2.3. The Modified d2

For the purpose of the present study, several modified versions of the d2-R test of sustained attention were created in E-Prime 2.0 [38]. All of these versions presented the letter “d” or “p” with one to four marks above and below it. The test-taker’s task was to decide whether the respective letter was a target; that is, a “d” with a total of two marks, or a nontarget and to press the key “c” for nontargets (left index finger; the key was colored red) or “m” for targets (right index finger; the key was colored green) on a German QWERTZ computer keyboard. While this task remained the same throughout the different versions, we varied the presentation mode to systematically manipulate characteristic features of sustained attention tests, i.e., stimulus arrangement and pace. 

**Figure 1:**
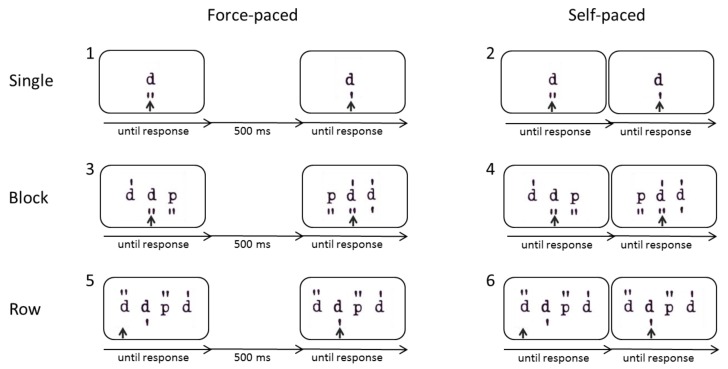
Illustration of the six different conditions of the modified d2, varying stimulus arrangement (single, block, row) and pace (force-paced [with response-stimulus interval] versus self-paced [without response-stimulus interval]). For all of these conditions, the task was to decide whether the relevant stimulus, as indicated by an arrow, was a d2 or not and to press the corresponding key.

Altogether, there were six blocks and each block consisted of 80 stimuli plus 10 practice trials. The whole task took about 15 minutes. In the first block, “single stimuli, force-paced”, a single letter (with marks) was presented at a time and the participant had to decide whether the respective letter was a d2 or not and press the respective key. After the test-takers’ response, there was a 500 ms response-stimulus interval (RSI) before the next letter appeared. This simple modified version of the d2 was created to measure the speed of the item solving process (*mental operation speed*) beyond perceptual and motor speed. Importantly, as single stimuli were presented and short intervals were included between successive stimuli, it did not require further critical mechanisms of sustained attention tests such as item shifting or preprocessing. In the second block, “single stimuli, self-paced”, a single letter was presented at a time but the response-stimulus interval was removed, so that this condition should require the immediate *shifting between items*. In the third block, “blocks of stimuli, force-paced”, three stimuli were presented at a time, but only the one in the center was relevant. After the test-taker made a response, there was a 500 ms response-stimulus interval before the next three letters appeared. Similarly, in the fourth block, “blocks of stimuli, self-paced”, three stimuli were presented at a time but this time, the response-stimulus interval was removed so that participants had to immediately *shift to the next item*. In the fifth block, “rows of stimuli, force-paced”, rows of stimuli were presented (ten stimuli at a time) and they became relevant one after another, allowing the *preprocessing* of upcoming stimuli. An arrow indicated the currently relevant stimulus and after responding to it, the screen went blank for a 500 ms response–stimulus interval until the stimuli appeared again and the arrow moved on to the next stimulus. Similarly, in the sixth block “rows of stimuli, self-paced”, rows of stimuli (ten stimuli at a time) were presented which became relevant successively and, therefore, allowed *preprocessing*. However, this time, the response-stimulus interval was removed so that the arrow moved on to the next item right after the test-takers’ response, requiring the test-taker to immediately shift to the next item. This final condition was designed to closely resemble the original presentation mode of the d2-R test of sustained attention (see Figure 1 for an illustration of the procedure). 

#### 2.2.4. Test d2-R Electronic Version [15]

The d2-R (electronic version) was applied as a conventional measure of sustained attention test performance. In the d2-R, the task is to select the letter “d” with a total of two marks out of many “d”s and “p”s with one to four marks by clicking on the respective letter with a computer mouse. 14 different screens with sixty letters per screen (structured in six rows of ten letters per row) were presented and the participants had to mark as many d2s as possible within 20 seconds. The dependent variable was the number of correctly marked items minus the number of confusion errors (error-corrected speed). Altogether, the task took 4.40 minutes plus instructions. 

### 2.3. Procedure

Participants were tested in groups of two to five in a laboratory. Each test session took about two hours and included two ten-minute breaks. The experiment started with a short questionnaire and cognitive tests irrelevant to the present study. After the first break, the inspection time task, the simple reaction time task, the modified d2 and further cognitive tasks were administered and the participants took another ten-minute break. The second break was followed by the administration of the electronic version of the d2-R and further cognitive tasks. Then, 30 minutes after the first administration of the inspection time task, the simple reaction time task and the modified d2, the participants performed these tasks a second time. Finally, they completed a short questionnaire to assess strategies and problems during the test session. 

### 2.4. Data Preprocessing

Participants who stated difficulties with the inspection time task in the post-experimental questionnaire and whose inspection time was above *z* = 4 were excluded as outliers (three participants in the first, two in the second administration). Participants whose accuracy in the modified d2 was below 70% were excluded from the analysis (three participants in the first, none in the second administration). Additionally, an accuracy below 70% in one of the conditions of the modified d2 led to an exclusion of the respective condition (three participants in the self-paced condition with rows of stimuli for both administrations). 

For the analysis of reaction times (RT) in the modified d2, errors were excluded (first administration: 5.3%, second administration: 5.3%) and for the correct trials, z-values were calculated for each participant in each block. Trials with RT above *z* = 2.5 and below *z* = −2.5 were excluded as outliers (first administration: 3.1%; second administration: 3.0%). The same cut-off values were applied for outliers in the simple reaction time task (first administration: 6.9%; second administration: 7.8%)^1^.

### 2.5. Analysis Strategy

Firstly, in order to identify the locus of the practice effect within the proposed process model, we investigated which of the tasks that were applied to measure the sub-components of sustained attention test performance, namely *perceptual, mental operation* or *motor speed* benefitted from practice and to which extent. Therefore, we computed the size of the practice effects in the respective tasks—that is, the practice effect in the inspection time task, the simple reaction time task, and the simple modified d2. 

Secondly, we turned towards further postulated mechanisms of sustained attention test performance; *item shifting* and *preprocessing*. It was investigated to what extent they benefitted from practice. Therefore, for RT and error rates, a repeated-measures ANOVA with the within-subject factors practice (first versus second administration) and pace (self-paced versus force-paced), as well as practice (first versus second administration) and stimulus arrangement (single versus blocks vs. rows of stimuli) was conducted and it was examined whether the stimulus arrangement or pace interacted with the practice of the task. For an effect of practice on *item shifting*, there should be an interaction of practice and pace so that the difference between the self-paced and the force-paced mode became smaller through practice. With regard to *preprocessing*, there should be an interaction of practice and stimulus arrangement, indicating that the preview benefit became larger through practice. 

## 3. Results

### 3.1. Descriptive Statistics and Practice Effects

Table 1 provides the means, standard deviations, the practice effects in ms, the effect sizes of the practice effects (Cohen’s d_z_) and the retest reliabilities of the tasks assessing the sub-components of the process model. In order to investigate the effects of practice on the sub-components, a repeated-measures ANOVA with the within-subjects variables practice (first versus second administration) and task (inspection time task, simple reaction time task versus simple modified d2) was conducted. There was a main effect of practice, *F*(1, 91) = 234.421, *p* < .001, η²_p_ = .720, demonstrating a strong practice effect in the three subtasks. Additionally, significant practice effects were confirmed for each of the three tasks by conducting separate t-tests (*p* < .01). Moreover, there was a main effect of the task itself, *F*(1.339, 121.890) = 4574.412, *p* < .001, η²_p_ = .980 2 and an interaction of practice and the task, *F*(1.401, 127.473) = 195.263, *p* < .001, η²_p_ = .682. Of the three tasks, the simple modified d2 benefitted most from practice (measure of *mental operation speed* beyond perceptual and motor processes; *M* = −97 ms), followed by considerably smaller practice effects in the simple reaction time task (measure of *motor speed* beyond basic perceptual processes; *M* = −12 ms) and the inspection time task (measure of *perceptual speed*; *M* = −6 ms). 

For the assessment of the sub-components, the selection of the tasks was based on the assumption that they would successively include additional sub-components of sustained attention tests (that is, the inspection time task as a measure of *perceptual speed*, the simple reaction time task as a measure of *motor speed* and basic perceptual processes, and the modified d2 as a measure of *mental operation speed* beyond perceptual and motor processes). Please note that the following analyses are based on interindividual differences rather than means: For stepwise multiple regression analyses displaying the incremental validity of these tasks for the prediction of performance in the original d2-R test of sustained attention [15], see Appendix A Table A1. In line with the assumption, there was an incremental increase in explained variance for each task that was applied to measure an additional sub-component: At t1 (t2), the inspection time task explained 7% (9%) of the d2-R variance. When the simple reaction time task was included in the model, it added another 7% (3%), and finally, the inclusion of the modified d2 increased the explained variance by 34% (29%).

### 3.2. Effects of Practice on Item Shifting and Preprocessing

#### 3.2.1. RT Analysis

**Figure 2:**
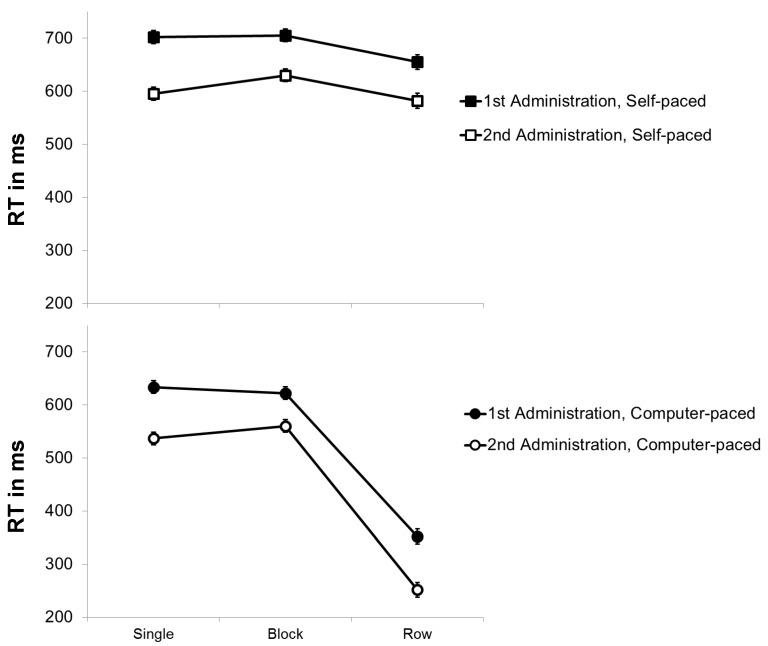
Mean RT (in ms) of the modified d2 as a function of stimulus arrangement (single versus blocks versus rows of stimuli) and pace (self-paced versus force-paced) for the first and second administration. Bars represent standard errors.

In order to assess the effects of practice on *item shifting*, a repeated-measures ANOVA with the within-subject variables *practice* (first versus second administration) and *pace* (self-paced versus force-paced) was conducted for RT in the different conditions of the modified d2 (see Figure 2). As expected, it revealed a significant effect of *practice*, *F*(1, 93) = 457.033, *p* < .001, η²_p_ = .831, confirming that RT decreased from the first (*M* = 611 ms) to the second (*M* = 521 ms) administration. Additionally, there was a significant effect of *pace*, *F*(1, 93) = 2186.440, *p* < .001, η²_p_ = .959. That is, the self-paced mode (*M* = 642 ms) impeded performance compared to the force-paced presentation of items (*M* = 490 ms). Finally, there was no significant *interaction between practice and pace*, *F*(1, 93) = .000, *p* = .990, η²_p_ = .000, indicating that the difference between the self-paced and the force-paced mode did not significantly change from the first (*M*_RT Difference_ = 152 ms) to the second administration (*M*_RT Difference_ = 152 ms). Thus, the results do not suggest that item shifting benefitted from practice. 

A repeated-measures ANOVA with the within-subject variables *practice* (first versus second administration) and *stimulus arrangement* (single, block versus row) was run to investigate the effects of practice on *preprocessing*. Besides the main effect of *practice*, the main effect of *stimulus arrangement* was significant, *F*(2, 186) = 643.587, *p* < .001, *η*²_p_ = .874. Post hoc tests using the Bonferroni correction showed that RT to rows of stimuli (*M* = 457 ms) were significantly shorter than RT to single (*M* = 614 ms) or blocks of stimuli (*M* = 628 ms), *p* < .001, revealing a strong *preview benefit*. Moreover, the interaction of practice and stimulus arrangement is crucial to assess the effects of practice on this preview benefit, since preview is restricted to the presentation of rows of stimuli. The interaction was significant, *F*(2, 186) = 19.411, *p* < .001, η²_p_ = .173. However, post hoc tests using the Bonferroni correction showed that the practice effect was larger for the presentation of single stimuli (*mean practice effect* = 105 ms) than for the presentation of rows of stimuli (*mean practice effect* = 93 ms), which then again was larger than the practice effect for blocks of stimuli (*mean practice effect* = 72 ms). Only the conditions presenting rows of stimuli allowed preprocessing but the practice effect in these conditions was smaller than the practice effect in the conditions with single stimuli, which did not allow preprocessing. Altogether, the data do not indicate that practice led to a substantially enlarged preview benefit.^3^

#### 3.2.2. Error Rates

**Figure 3:**
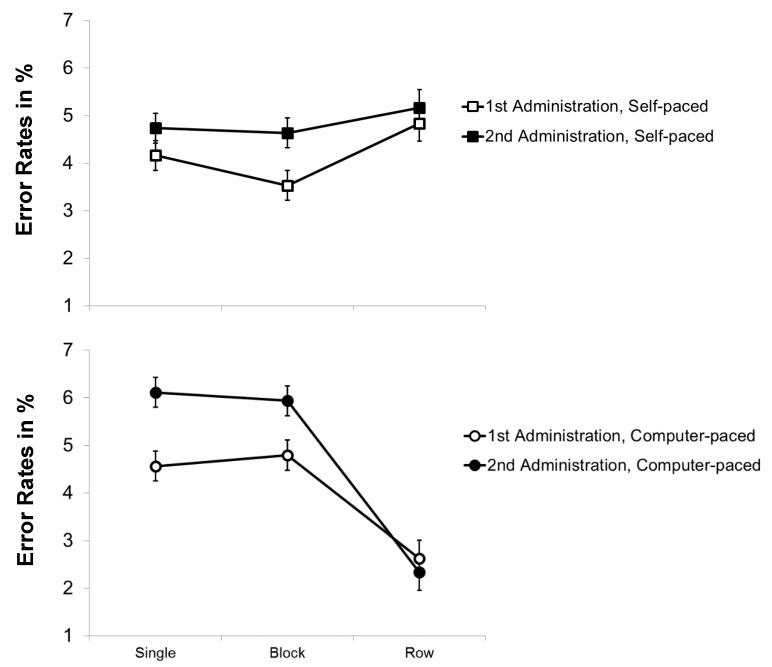
Mean error rates (in%) of the modified d2 as a function of stimulus arrangement (single versus blocks versus rows of stimuli) and pace (self-paced versus force-paced) for the first and second administration. Bars represent standard errors.

Error rates were generally low (2.3–6.1%) and the analyses yielded small effect sizes for the main effects and interactions (see Figure 3). The repeated-measures ANOVA with the within-subjects variables *practice* and *pace* revealed a significant main effect of *practice*, *F*(1, 93) = 10.850, *p* = .001, η²_p_ = .104. Surprisingly, error rates were slightly higher for the second administration of the modified d2 (*M* = 4.6%) than for the first (*M* = 4.1%), indicating a speed-accuracy trade-off in the second administration. The main effect of *pace* and the interaction of *practice* and *pace* were not significant, *F*(1, 93) < 1, *p* > .3, η²_p_ < .02. Thus, the results do not indicate that *item shifting* benefitted from practice with respect to accuracy.

For the repeated-measures ANOVA with the within-subjects variables *practice* and *stimulus arrangement*, besides the main effect of *practice*, there was also a main effect of *stimulus arrangement*, *F*(2, 186) = 16.663, *p* < .001, η²_p_ = .152. Post hoc tests using the Bonferroni correction showed that error rates were significantly lower for the conditions with rows of stimuli (*M* = 3.7%) than for the conditions with single (*M* = 4.8%) or blocks of stimuli (*M* = 4.6%, *p* < .001), indicating a *preview benefit* for rows of stimuli. Finally, there was a significant interaction of *practice* and *stimulus arrangement*, *F*(2, 186) = 7.448, *p* = .001, η²_p_ = .074. This interaction showed that, for the presentation of single and blocks of stimuli, error rates increased from the first to the second administration (1st *M*_single_ = 4.3% and *M*_blocks_ = 4.1%, 2nd *M*_single_ = 5.2% and *M*_blocks_ = 5.1%) while they stayed roughly the same for rows of stimuli (1st *M*_rows_ = 3.8%, 2nd *M*_rows_ = 3.6%). Altogether, the data do not indicate that error rates decreased with practice for the presentation of rows of stimuli, suggesting that the preview did not markedly benefit from practice. 

## 4. Discussion

The aim of the present study was to explore the locus of the practice effect in sustained attention tests from two different angles of approach: First, drawing on a recently proposed process model of sustained attention tests, we investigated which of the subcomponents of performance, namely *perceptual, mental operation* and *motor speed*, benefitted from practice and to which extent. Second, we looked into further mechanisms that have been shown to play a role in sustained attention tests, namely *item shifting* and *preprocessing*, and examined whether they became more efficient through practice. Subtasks that assessed the speed in the sub-components of sustained attention tests and modified versions of the d2 test of sustained attention were applied twice to obtain measures of the respective sub-components and the *item shifting* and *preprocessing* mechanisms in the first and second test administration. 

With regard to the process model, it was shown that all the three tasks that assessed the sub-components of sustained attention tests, i.e., the *perception* of the item, a *simple mental operation* to solve the item, and the *motor reaction*, benefitted from practice. However, while effect sizes of the practice effect were small for the measures of *perceptual* and *motor speed*, the practice effect was large for the task that additionally required item solving processes. Thus, the two additional demands involved in this task—that is, the *simple mental operation* to solve the item and possibly also the *coordination* of the three sub-components—seem to especially benefit from practice. Regarding the preprocessing of upcoming items and the deliberate shifting between items, the results do not support a practice effect for these postulated mechanisms of sustained attention tests. Thus, the large practice effect in sustained attention tests cannot be attributed to a more efficient *item shifting* or *preprocessing*.

### 4.1. Effects of Practice on the Sub-Components

The first aim of the present study was to explore which of the sub-components of sustained attention tests benefitted from practice and could therefore cause the large practice effect in this group of tests. All of the tasks assessing the sub-components of the process model showed practice gains. However, effect sizes were rather small for the inspection time and simple reaction time task, which were applied to obtain measures of *perceptual* [32] and *motor* speed [35]. That means that getting used to the stimulus material and the input device, which leads to a more efficient *perception* and *motor reaction* enhances the test-takers’ performance only to a limited extent. However, it is important to note that our sample was young and very skilled in using a computer keyboard. Thus, with regard to motor speed, it is conceivable that the practice effect is considerably larger for elderly test-takers who are less used to a computer keyboard.

Strikingly, the practice effect was large for the simple modified d2, which involved all of the three sub-components of sustained attention tests, namely perceptual, mental operation and motor speed. The size of the practice effect in this task suggests that the additional requirements of this task—that is, the speeded processing and decision on the correct answer to the item—seem to especially benefit from practice. Hence, the relatively basic decisions required in sustained attention tests (e.g., deciding whether a stimulus is a d2 or not or whether numbers add up or not) seem to become more efficient through the repeated administration and to speed up reactions substantially. 

Beyond that, this task also imposed higher demands on the coordination of action patterns than the other tasks. The concept of coordination—that is, the organization, efficient timing and execution of action patterns—has long been considered a key demand of sustained attention tests [1,40,41]. As the simple modified d2 required all of the three sub-components, it also imposed higher demands on their coordination than the less complex measures of perceptual and motor speed. Hence, when the responses to the simple modified d2 speeded up this could partly be due to a more efficient coordination of the sub-components. In an earlier study, Krumm et al. [41] demonstrated an influence of the coordination of action patterns on the practice effect in a sustained attention test. 

### 4.2. Effects of Practice on Preprocessing and Item Shifting

The second aim of the present study was to investigate whether two postulated mechanisms in sustained attention tests, namely *item shifting* and *preprocessing*, benefitted from practice and could therefore cause the large practice effect in these tests. In order to do so, a modified version of the d2-R test of sustained attention was created in which the pace (self-paced versus force-paced) was varied so that it either required the deliberate, self-paced *shifting between items* (self-paced conditions) or did not require such a thing (force-paced conditions). Additionally, the stimulus arrangement (single blocks versus rows of stimuli) was varied to not allow (single or blocks of stimuli) versus to allow *preprocessing* (rows of stimuli, which became successively relevant), respectively. These different conditions of the modified d2 were administered twice in order to explore whether *item shifting* or *preprocessing* became more efficient through practice. 

However, the data did not indicate that practice had a positive effect on either *item shifting* or *preprocessing*. Regarding *item shifting*, there was no significant interaction of practice and pace and the difference between the self-paced and the force-paced condition remained largely unchanged throughout both administrations. With regard to *preprocessing*, while there was an interaction of practice and stimulus arrangement, it indicated that the practice effect for single stimuli (which did not allow preprocessing) was even larger than for rows of stimuli (which allowed preprocessing), suggesting that practice did not reinforce preprocessing. Indeed, the relative size of the preview benefit effect remained the same throughout both administrations. Altogether, while the present data reflected the role of *item shifting* and *preprocessing* in sustained attention tests, they did not indicate that either of these mechanisms benefitted from practice. Quite the contrary, these results suggest that the large practice effect in sustained attention tests is most likely not due to a more efficient *item shifting* or *preprocessing* in retest.

### 4.3. Limitations and Strenghts

It is important to acknowledge the limitations of the present study. First, the present sample was not representative with regard to age, sex or educational level and the present findings cannot be generalized to other samples. That is, our sample mostly consisted of young and female students, who are, for example, typically very skilled in using a computer keyboard. It is therefore conceivable that the small practice effect in the simple reaction time task was due to a ceiling effect. Moreover, the time interval between the two test administrations was rather short (30 min), so the present results cannot be easily generalized to longer test–retest intervals. 

Furthermore, there was a small speed–accuracy trade-off with regard to the practice effect in the modified d2. That is, while reaction times considerably decreased from the first to the second administration, there was a slight increase in the error rates. Thus, it seems that the practice effect in the second administration of the modified d2 was partly due to a style of working that focused more on speed than on accuracy. However, it is important to note that the size of the reversed practice effect in the error rates was descriptively small (error rates of 4.1% in the first and 4.6% in the second administration) and also moderate in its effect size (*η²_p_* = .104), while the practice effect in the reaction times was very large (1st administration: *M* = 611 ms; 2nd administration: *M* = 521 ms; *η²_p_* = .831).

Finally, we investigated the effects of practice on the sub-components of sustained attention tests using measures of *perceptual*, *mental operation* and *motor speed*. More specifically, we selected cognitive tasks so that they would successively demand additional sub-components (e.g., the inspection time task as a measure of perceptual speed, the simple reaction time task as a measure of motor speed and basic perceptual processes, and the modified d2 as a measure of mental operation speed beyond perceptual and motor processes). However, these sub-components are not necessarily strictly independent or discrete [42,43,44]. We therefore might have not fully disentangled the specific influences of the different sub-components.

Despite these weaknesses, a major advantage of the present study was the systematic application of different tasks and the experimental manipulation of important task features that are postulated to influence performance in sustained attention tests. Using an experimental test validation approach, we were able to examine different potential causes of practice effects in sustained attention tests and altogether, to gain a deeper understanding of the mechanisms that underly performance in these tasks (see also [45,46]).

### 4.4. Conclusions

The present study set out to explore the locus of the large practice effect in sustained attention tests. Our study suggests that item solving processes and possibly also the coordination of *perceptual, mental operation* and *motor* processes seem to benefit most from practice, while the practice effects are much smaller for *perceptual* and *motor* components. As there are many different sustained attention tests that demand different mental operations (e.g., letter cancellation, mental arithmetic, and sorting according to categories), it would be interesting to systematically investigate whether some of these item-solving processes are less susceptible to practice than others and why that is the case. Moreover, it would be interesting to further examine how practice effects in sustained attention tests relate to practice effects in other cognitive ability tests. Generally, it has again been shown that the field of psychological assessment greatly profits from experimental methods because they allow a closer look into phenomena like the large practice effect in cognitive ability tests, which is imperative to address or tackle them. We hope that the present paper has taken a first step towards a deeper understanding of practice effects in sustained attention tests.

## Figures and Tables

**Table 1:** Mean reaction times/inspection time (in ms), standard deviations, retest reliabilities and practice effects (in ms) of the complex modified d2 and the tasks assessing the sub-components of sustained attention test performance.

Tasks (Sub-Components)	1st Administration	2nd Administration	Practice Effect				Reliability
	*M (SD)*	*M (SD)*	*T*	*p*	*M (SD)*	*d_z_*	*r_tt_*
Inspection Time Task (perceptual speed)	53.73 (27.76)	47.35 (31.83)	3.14	<.01	6.38 (26.00)	0.32	.58
Simple Reaction Time Task (motor speed)	239.60 (26.27)	227.43 (26.15)	5.01	<.001	12.17 (24.30)	0.50	.57
Simple modified d2 (mental operation speed)	634.00 (95.54)	536.95 (72.52)	16.88	<.001	97.05 (61.28)	1.71	.68

## Footnotes

1 An outlier analysis based on logarithmized RT yielded very similar results.

2 Mauchly’s sphericity test indicated a violation of the sphericity assumption for the variable task (*χ2*(2) = 61.158, *p* < .001) and for the interaction of practice and task (*χ2*(2) = 50.237, *p* < .001). Therefore, the Greenhouse–Geisser correction was used to adjust degrees of freedom for the main effect of task (ε = 0.670) and for the interaction of practice and task (ε = 0.700).

3 Additional analyses revealed that practice effects within sessions were less systematic than practice effects between sessions, which is most likely due to the role of memory consolidation for practice effects [39].

## Appendix A

**Table A1:** Performance in the original d2-R test of sustained attention regressed on the tasks representing the sub-components of sustained attention tests (stepwise multiple regression).

Predictors	1st Administration				2nd Administration			
	*β*	R^2^	adj. R^2^	ΔR^2^	*β*	R^2^	adj. R^2^	ΔR^2^
Model 1								
Inspection Time Task (perceptual speed)	.26 *	.07 *	.06		.31 **	.09 **	.09	
Model 2								
Inspection Time Task (perceptual speed)	.23 *				.26 *			
Simple Reaction Time Task (motor speed)	.26 *	.13 **	.11	.07 *	.18 *	.13 **	.11	.03 *
Model 3								
Inspection Time Task (perceptual speed)	.08				.09			
Simple Reaction Time Task (motor speed)	.02				.02			
Simple Modified d2 (mental operation speed)	.67 **	.47 **	.46	.34 **	.60 **	.41 **	.40	.29 **

## References

[1] Westhoff K., Hagemeister C. (2005). Konzentrationsdiagnostik.

[2] Brickenkamp R., Schmidt-Atzert L., Liepmann D. (2010). Test D2-Revision. Aufmerksamkeits-Und Konzentrationstest (D2-R).

[3] Mirsky A.F., Anthony B.J., Duncan C.C., Ahearn M.B., Kellam S.G. (1991). Analysis of the Elements of Attention: A Neuropsychological Approach. Neuropsychol. Rev..

[4] Schmidt-Atzert L., Bühner M., Schweizer K. (2000). Aufmerksamkeit Und Intelligenz. Intelligenz und Kognition: Die Kognitiv biologische Perspektive der Intelligenz.

[5] Schmidt-Atzert L., Bühner M., Enders P. (2006). Messen Konzentrationstests Konzentration? Eine Analyse Der Komponenten von Konzentrationsleistungen. Diagnostica.

[6] Büttner G., Schmidt-Atzert L. (2004). Diagnostik von Konzentration Und Aufmerksamkeit.

[7] Bühner M., Ziegler M., Bohnes B., Lauterbach K. (2006). Übungseffekte in Den TAP Untertests Test Go/Nogo Und Geteilte Aufmerksamkeit Sowie Dem Aufmerksamkeits-Belastungstest (D2). Z. Für Neuropsychol..

[8] Lievens F., Reeve C.L., Heggestad E.D. (2007). An Examination of Psychometric Bias Due to Retesting on Cognitive Ability Tests in Selection Settings. J. Appl. Psychol..

[9] Randall J.G., Villado A.J. (2017). Take Two: Sources and Deterrents of Score Change in Employment Retesting. Hum. Resour. Manag. Rev..

[10] Scharfen J., Peters J.M., Holling H. (2018). Retest Effects in Cognitive Ability Tests: A Meta-Analysis. Intelligence.

[11] Hausknecht J.P., Halpert J.A., Di Paolo N.T., Moriarty Gerrard M.O. (2007). Retesting in Selection: A Meta-Analysis of Coaching and Practice Effects for Tests of Cognitive Ability. J. Appl. Psychol..

[12] Krumm S., Schmidt-Atzert L., Michalczyk K., Danthiir V. (2008). Speeded Paper-Pencil Sustained Attention and Mental Speed Tests. J. Individ. Differ..

[13] Westhoff K., Dewald D. (1990). Effekte Der Übung in Der Bearbeitung von Konzentrationstests. Diagnostica.

[14] Steinborn M.B., Langner R., Flehmig H.C., Huestegge L. (2017). Methodology of Performance Scoring in the D2 Sustained-Attention Test: Cumulative-Reliability Functions and Practical Guidelines. Psychol. Assess..

[15] Schmidt-Atzert L., Brickenkamp R. (2017). Test D2-R-Elektronische Fassung Des Aufmerksamkeits-Und Konzentrationstests D2-R.

[16] Scharfen J., Blum D., Holling H. (2018). Response Time Reduction Due to Retesting in Mental Speed Tests: A Meta-Analysis. J. Intell..

[17] Te Nijenhuis J., van Vianen A.E.M., van der Flier H. (2007). Score Gains on G-Loaded Tests: No G. Intelligence.

[18] Lievens F., Buyse T., Sackett P.R. (2005). Retest Effects in Operational Selection Settings: Development and Test of a Framework. Pers. Psychol..

[19] Westhoff K., Graubner J. (2003). Konstruktion Eines Komplexen Konzentrationstests. Diagnostica.

[20] Krumm S., Schmidt-Atzert L., Schmidt S., Zenses E.M., Stenzel N. (2012). Attention Tests in Different Stimulus Presentation Modes. J. Individ. Differ..

[21] Krumm S., Schmidt-Atzert L., Eschert S. (2008). Investigating the Structure of Attention: How Do Test Characteristics of Paper-Pencil Sustained Attention Tests Influence Their Relationship with Other Attention Tests?. Eur. J. Psychol. Assess..

[22] Blotenberg I., Schmidt-Atzert L. (2019). Towards a Process Model of Sustained Attention Tests. J. Intell..

[23] Blotenberg I., Schmidt-Atzert L. (2019). On the Characteristics of Sustained Attention Test Performance—The Role of the Preview Benefit. Eur. J. Psychol. Assess..

[24] Schweizer K., Moosbrugger H. (2004). Attention and Working Memory as Predictors of Intelligence. Intelligence.

[25] Van Breukelen G.J.P., Roskam E.E.C.I., Eling P.A.T.M., Jansen R.W.T.L., Souren D.A.P.B., Ickenroth J.G.M. (1995). A Model and Diagnostic Measures for Response Time Series on Tests of Concentration: Historical Background, Conceptual Framework, and Some Applications. Brain Cognit..

[26] Eriksen C.W. (1995). The Flankers Task and Response Competition: A Useful Tool for Investigating a Variety of Cognitive Problems. Vis. Cognit..

[27] Friedman N.P., Miyake A. (2004). The Relations Among Inhibition and Interference Control Functions: A Latent-Variable Analysis. J. Exp. Psychol. Gen..

[28] Rayner K. (1998). Eye Movements in Reading and Information Processing: 20 Years of Research. Psychol. Bull..

[29] Schotter E.R., Angele B., Rayner K. (2012). Parafoveal Processing in Reading. Atten. Percept. Psychophys..

[30] Brand C.R., Deary I.J., Eysenck H. (1982). Intelligence and “Inspection Time.” In A Model of Intelligence.

[31] Schweizer K., Koch W. (2003). Perceptual Processes and Cognitive Ability. Intelligence.

[32] Vickers D., Nettelbeck T., Willson R.J. (1972). Perceptual Indices of Performance: The Measurement of ‘Inspection Time’ and ‘Noise’ in the Visual System. Perception.

[33] Ackerman P.L. (1988). Determinants of Individual Differences during Skill Acquisition: Cognitive Abilities and Information Processing. J. Exp. Psychol. Gen..

[34] Fleishman E.A., Hempel W.E. (1954). Changes in Factor Structure of a Complex Psychomotor Test as a Function of Practice. Psychometrika.

[35] Sternberg S., Monsell S., Knoll R.L., Wright C.E., Stelmach G.E. (1978). The Latency and Duration of Rapid Movement Sequences: Comparisons of Speech and Typewriting. Information Processing in Motor Control and Learning.

[36] Neubauer A.C., Knorr E. (1997). Elementary Cognitive Processes in Choice Reaction Time Tasks and Their Correlations with Intelligence. Pers. Individ. Dif..

[37] Vickers D., Smith P.L. (1986). The Rationale for the Inspection Time Index. Pers. Individ. Dif..

[38] Schneider W., Eschman A., Zuccolotto A. (2002). E-Prime 2.0 Software.

[39] McGaugh L.J. (1966). Time-Dependent Processes in Memory Storage. Science.

[40] Düker H. (1957). Leistungsfähigkeit Und Keimdrüsenhormone.

[41] Krumm S., Schmidt-Atzert L., Bracht M., Ochs L. (2011). Coordination as a Crucial Component of Performance on a Sustained Attention Test. J. Individ. Differ..

[42] Townsend J.T. (1990). Serial vs. Parallel Processing: Sometimes They Look like Tweedledum and Tweedledee but They Can (and Should) Be Distinguished. Psychol. Sci..

[43] Schubert A.-L., Hagemann D., Voss A., Schankin A., Bergmann K. (2015). Decomposing the Relationship between Mental Speed and Mental Abilities. Intelligence.

[44] Stafford T., Gurney K.N. (2011). Additive Factors Do Not Imply Discrete Processing Stages: A Worked Example Using Models of the Stroop Task. Front. Psychol..

[45] Bornstein R.F. (2011). Toward a Process-Focused Model of Test Score Validity: Improving Psychological Assessment in Science and Practice. Psychol. Assess..

[46] Borsboom D., Mellenbergh G.J., Van Heerden J. (2004). The Concept of Validity. Psychol. Rev..

